# Adsorption of Methylene Blue onto Environmentally Friendly Lignocellulosic Material Obtained from Mature Coltsfoot (*Tussilago farfara*) Leaves

**DOI:** 10.3390/polym17111549

**Published:** 2025-06-02

**Authors:** Giannin Mosoarca, Cosmin Vancea, Simona Popa, Maria Elena Radulescu-Grad, Mircea Dan, Cristian Tanasie, Sorina Boran

**Affiliations:** 1 Faculty of Chemical Engineering, Biotechnologies and Environmental Protection, Politehnica University Timisoara, V. Parvan Bd. No. 6, 300223 Timisoara, Romania; giannin.mosoarca@upt.ro (G.M.); cosmin.vancea@upt.ro (C.V.); simona.popa@upt.ro (S.P.); mircea.dan@upt.ro (M.D.); 2 Coriolan Dragulescu Institute of Chemistry, Romanian Academy, Mihai Viteazu Bd. No. 24, 300223 Timisoara, Romania; 3 National Institute of Research and Development for Electrochemistry and Condensed Matter (INCEMC), Dr. A. Paunescu Podeanu St., no.144, 300569 Timisoara, Romania; tase@incemc.ro

**Keywords:** adsorption, methylene blue, coltsfoot (*Tussilago farfara*), equilibrium isotherm, kinetic model, Taguchi method

## Abstract

The present study investigates the potential of a new lignocellulosic adsorbent material obtained from mature coltsfoot (*Tussilago farfara*) leaves for the removal of methylene blue from aqueous solutions. The material was obtained after minimal processing of the leaves, without chemical or thermal treatment. The material was first characterized using several specific techniques (FTIR, color analysis). Then, the mechanism of the adsorption process was investigated through studies related to adsorption equilibrium, kinetics, and thermodynamics. The adsorption process is described by the Sips isotherm and the general kinetic model, while the thermodynamic parameters suggest that physical adsorption is the primary mechanism responsible for dye retention. The Taguchi method was used to optimize the adsorption conditions and to identify the most influential controllable factor. ANOVA was used to calculate the percentage contribution of each controllable factor to the dye removal efficiency. pH had the greatest influence on the process (87.78%), while temperature had the least effect (0.16%). The maximum adsorption capacity determined was 278.1 mg/g, being higher than other similar adsorbents. All the results show that coltsfoot (*Tussilago farfara*) leaves are a very cheap, environmentally friendly, and effective adsorbent for the removal of methylene blue dye from aqueous solutions.

## 1. Introduction

As industrialization accelerates and populations expand, pollution from factories and households has worsened considerably, creating serious detriments to economic progress, urban development, and public health. The industrial textile sector extensively uses organic colorants for dyeing, particularly cationic dyes prized for their vibrant hues and resistance to fading and water damage. Nevertheless, the effluent generated during the production and application of these dyes is characterized by intense coloration, poor light penetration, and considerable persistence, severely hindering the natural cleansing processes of water bodies and presenting a grave threat to ecosystems and aquatic organisms. Therefore, exploring efficacious techniques for the thorough elimination of these dyes from wastewater is crucial [1,2].

Beyond its function as a colorant in the textile sector, methylene blue also finds utility in medicine or in treating fungal infections within fish farming. Although this compound offers various advantageous uses, it can also induce unfavorable reactions in people, including cephalalgia, stomach inflammation, queasiness, and elevated pulse, and provoke dermal discomfort and transient or lasting ocular damage [3,4].

Therefore, a fundamental step involves the proper treatment of wastewater originating from the textile industry before its release into the natural environment. Various chemical and physical processes have been employed to remove dyes from liquid effluents, including oxidation, electrolysis, sedimentation, ion exchange, coagulation–flocculation, membrane separation technology, and biological treatment [5,6,7]. Recently, new techniques have been developed such as the use of emerging oxidants to produce free radicals or auto-Fenton photocatalysis processes to remove organic pollutants, including organic dyes, from water [8,9]. However, these approaches present significant disadvantages and limitations in their application, as they can be costly, inefficient, and generate considerable quantities of solid waste, which not only seriously threatens the environment but also entails high financial expenditures, cost being the main disadvantage [3,10].

In contrast to other methods, adsorption presents itself as an economical, environmentally sound, and industrially scalable alternative for treating dye-contaminated water, efficiently diminishing their adverse impacts on ecosystems. Indeed, adsorption has surfaced as the favored approach for intricate wastewater systems owing to its notable efficiency and adaptability. This technique not only excels at removing dyes but also concurrently eliminates a spectrum of pollutants, encompassing organic compounds and heavy metal ions. Considering its operational simplicity, cost-effectiveness, and ecological compatibility, adsorption technology constitutes a preferred solution for a wide array of application contexts [3,11,12,13,14].

To achieve adsorption, a variety of adsorbents can be used, such as carbon-based, mineral, magnetic, metal–organic, agricultural waste, and others. Since the cost of the adsorbent material greatly influences the total cost of the process (approximately 70%), researchers are motivated to identify accessible materials from industrial and agricultural wastes, minerals, and plant sources. This need has led to the exploration of inexpensive adsorbent materials, which can be used directly or after simple processing, such as agricultural wastes and natural plant materials [15,16,17]. Various adsorbents obtained from plant leaves have been successfully used to remove methylene blue from water, such as *Humulus japonicas* leaves [18], *Daucus carota* leaves [19], sour-cherry leaves [20], bilberry leaves [21], *Magnolia grandiflora* leaves [22], *Typha angustifolia* leaves [23], *Platanus orientalis* leaves [24], Ficcus Palmata leaves [25], Ginkgo biloba leaves [26], Salix babylonica leaves [27], phoenix tree leaves [28], and lotus leaf [29].

*Tussilago farfara* L., or coltsfoot, is a perennial plant of the *Asteraceae* family, living predominantly within the Earth’s temperate zones. Its ancestral habitat spans the European landmass, a considerable portion of the Asian continent and the northern reaches of Africa. Through human-mediated dispersal, it has naturalized in diverse regions, notably North America, the Indian subcontinent, and the Far Eastern territories. Coltsfoot is a non-woody plant with a history in traditional healing practices. Its age-old application is principally linked to the maintenance of respiratory well-being. Scientific analyses have already elucidated the presence of a diverse array of biochemical compounds within its leaves and immature flowers, including sesquiterpenoids, phenolic acids, flavonoids, and alkaloids, among others. The leaves are large, circular, heart-shaped, measuring 3 to 12 cm long and 4 to 14 cm in diameter at their widest point. Their vein pattern radiates outwards, and their edges are characterized by a wavy, slightly serrated appearance [30,31].

This investigation aimed to test a new adsorbent material, obtained by minimal processing of coltsfoot leaves, for the adsorption of methylene blue from water. The initial phase involved a thorough characterization of the adsorbent materials using FTIR and colorimetric techniques. Subsequently, the study explored the impact of key factors governing the adsorption of dyes. Furthermore, it included analyses of kinetics, equilibrium, thermodynamic properties, and process optimization.

## 2. Materials and Methods

### 2.1. Obtaining the Adsorbent Material

The adsorbent material was obtained by minimal processing (grinding, washing with distilled water, drying) of mature coltsfoot leaves, which were purchased from StefMar (Ramnicu Valcea, Romania). First, 50 g of dried mature leaves were ground into a fine powder with an electric mill (5 min) and passed through a 2 mm sieve. The obtained powder was washed with distilled water (one wash cycle in solid:liquid ratio = 1:10) to remove color and turbidity and then dried in an oven at 105 °C for 24 h.

### 2.2. Adsorbent Characterization

To evaluate the surface characteristics of the adsorbent, several techniques were used, such as FTIR analysis, color analysis (CIELab* system), and determination of the point of zero charge (pH_PZC_). For this purpose, the following devices were used: Shimadzu Prestige-21 FTIR spectrophotometer (Shimadzu, Kyoto, Japan), a Cary-Varian 300 Bio UV-VIS colorimeter (Varian Inc., Mulgrave, Australia). The point of zero charge (pH_PZC_) was determined by the solid addition method [19] using a WTW Ino-lab pH meter (model 7310, Xylem Analytics Germany, Weilheim, Germany).

### 2.3. Adsorption Experiments

All adsorption experiments were performed in batch mode, with each condition tested in triplicate. The key parameters influencing the process were varied, and the adsorption capacity values and the dye removal efficiency values were monitored. The parameters influencing the process were varied within the following ranges: pH from 2 to 10, contact time between 2 and 40 min, temperature ranging from 278 to 311 K, initial dye concentration from 25 to 500 mg/L, adsorbent dosage from 1 to 5 g/L, and ionic strength between 0 and 0.25 mol/L. pH adjustments were made using dilute solutions of HCl (0.1 M) and NaOH (0.1 M), while the ionic strength was controlled by adding solid NaCl. The concentration of methylene blue was quantified using a Specord 200 PLUS UV-VIS spectrophotometer (Analytik Jena, Jena, Germany) at a detection wavelength of 664 nm.

### 2.4. Equilibrium, Kinetics, and Thermodynamics

The adsorption equilibrium and kinetics were examined by fitting the experimental data to various adsorption isotherms and kinetic models. These isotherms and models, along with their respective non-linear equations, are provided in Table S1 of the Supplementary Materials [32,33]. To identify the most suitable isotherm and kinetic model, several parameters (R^2^, SSE, χ^2^,) were computed. The formulas for calculating these parameters can be found in Table S2 of the Supplementary Materials [33].

The primary adsorption mechanism was determined through the thermodynamic parameters, which were calculated using the equations provided in Table S3 of the Supplementary Materials [32].

### 2.5. Process Optimization

To optimize the key factors influencing the adsorption process and enhance the dye removal efficiency, the Taguchi method—a robust experimental design approach—was employed. For this, an L27 orthogonal array with six factors at three levels was utilized, and the signal-to-noise ratio (S/N) was analyzed to evaluate the experimental outcomes. ANOVA analysis was then applied to assess the Taguchi results and to determine the percentage contribution of each controllable factor to the dye removal efficiency. The necessary calculations were performed using Minitab 19 Software (version 19.1.1, Minitab LLC, State College, PA, USA).

## 3. Results and Discussion

### 3.1. Adsorbent Characterization

**Figure 1:**
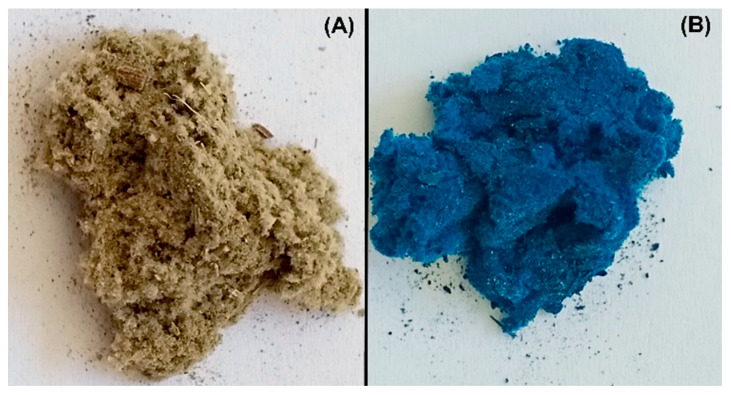
Appearance and color of the adsorbent material: (**A**) before adsorption, (**B**) after methylene blue adsorption.

Figure 1 shows the comparative appearance of the adsorbent material, obtained from coltsfoot leaves, before and after the adsorption of the dye. It can be seen that after adsorption, the material has a bluish color, which confirms the retention of methylene blue on its surface.

**Figure 2:**
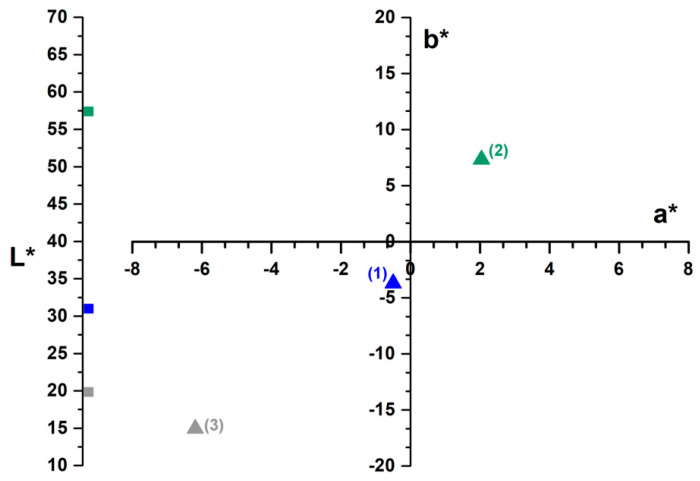
CIEL*a*b* color parameters of adsorbent, obtained from coltsfoot leaves, before and after adsorption of methylene blue: (**1**)—(MB) methylene blue, (**2**)—adsorbent before adsorption, (**3**)—adsorbent after adsorption.

Figure 2 presents the CIEL*a*b* color analysis results of the adsorbent material both prior to and following dye adsorption. The data indicate that the L*, a*, and b* values change after the adsorption of methylene blue. Point (1) corresponds to the color of methylene blue, and its color parameters indicate that the luminosity is low, and the color is green blue. Point (2) represents the color (light orange) of the adsorbent material, which has a higher luminosity. Point (1) corresponds to the color of methylene blue, and its color parameters indicate that the luminosity is low, and the color is green blue.

After adsorption, the luminosity decreases, the a* parameter moves from red to green and the b* parameter from yellow to blue, as a result, the adsorbent material is darker, and changes color to green blue, showing that the color parameters move to point (3) in the color space. This happened because the adsorbent material attracts and retains the methylene blue dye on its surface, leading to color uptake. As a result, the color is darker and shifts toward the region (quadrant) associated with the methylene blue color in the dyes quadrant.

Similar results have been reported in the scientific literature for the adsorption of methylene blue on sour-cherry leaves [20], bilberry leaves [21], and raspberry leaves [34].

**Figure 3:**
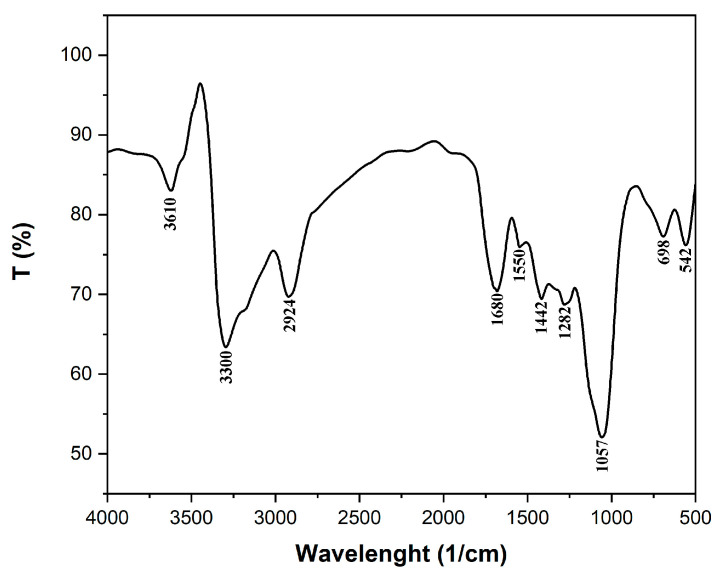
The FTIR spectrum for the adsorbent obtained from coltsfoot leaves (*Tussilago farfara* L.).

The FTIR spectrum for the adsorbent obtained from coltsfoot leaves is illustrated in Figure 3. It is well known that plant materials, such as plant leaves, have a ligno-cellulosic structure. The FTIR spectrum mainly highlights bands specific to some functional groups in the structure of cellulose, hemicellulose, and lignin. These bands are detailed in Table 1.

A key factor in adsorption research is the point of zero charge (pH_PZC_), which refers to the pH at which the net surface charge of an adsorbent becomes neutral. When the pH exceeds the pH_PZC_, the adsorbent surface carries a negative charge, promoting the adsorption of cationic dyes, due to the appearance of electrostatic attraction, whereas at lower pH values, the surface becomes positively charged, leading to the opposite effect [27,48].

**Figure 4:**
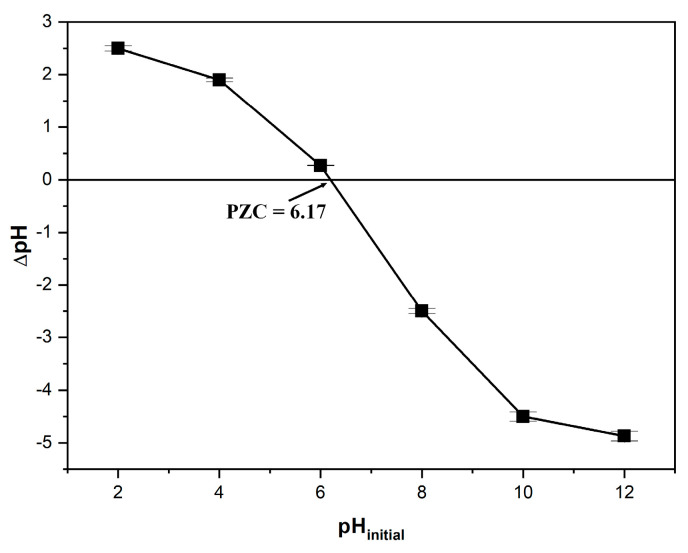
Determination of the zero point of charge (pH_PZC_) for the adsorbent obtained from coltsfoot leaves (*Tussilago farfara* L.) by the solid addition method.

The pH_PZC_ value for the adsorbent derived from coltsfoot leaves was found to be 6.1 (Figure 4).

### 3.2. Kinetic Study

**Figure 5:**
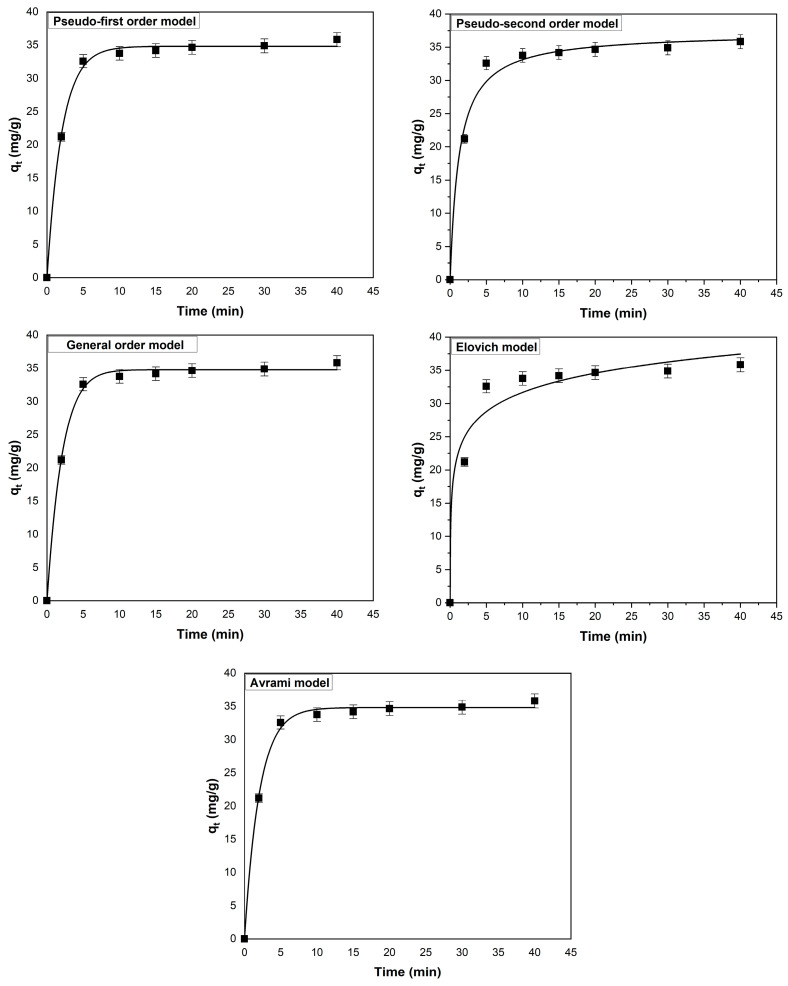
Experimental data points and fitted kinetic models for the adsorption of methylene blue on the adsorbent derived from coltsfoot leaves (experimental conditions: pH = 6, initial dye concentration = 50 mg/L, adsorbent dosage = 1 g/L, temperature = 295 K, ionic strength = 0 mol/L).

Figure 5 illustrates the effect of contact time on the adsorption capacity of the adsorbent obtained from coltsfoot leaves. The adsorption capacity increases until reaching equilibrium at 20 min. Initially, the increase is rapid, but with the passage of time, the rate of increase slows down, eventually reaching equilibrium. This pattern can be attributed to the availability of a large number of adsorption sites in the early stages, which are progressively filled until the adsorbent surface is almost completely covered with methylene blue molecules, signifying the equilibrium point [49,50].

Table 2 presents a comparison of the time required to achieve equilibrium in the adsorption of methylene blue on different plant-leaf-derived adsorbents.

The time-dependent adsorption data were analyzed using five kinetic models: pseudo-first order, pseudo-second order, general order model, Elovich model, and Avrami model. The kinetic curves for these models (based on non-linear equations provided in Table S1 of the Supplementary Materials), along with their fit to the experimental results, respectively, are shown in Figure 5. The calculated constants for these models and associated error parameters are presented in Table 3. The values of determination coefficient (R^2^), sum of square error (SSE), chi-square (χ^2^), and average relative error (ARE) suggest that the general order kinetic model most accurately represents the adsorption process. This kinetic model had the highest value for R^2^ and the lowest values for SSE, χ^2^, and ARE.

Similar results were reported in the scientific literature at methylene blue adsorption on similar adsorbents: bilberry (*Vaccinium myrtillus* L.) leaves [21], *Leonurus cardiaca* L. biomass [51], and *Handroanthus albus* bark [52].

### 3.3. Equilibrium Study

**Figure 6:**
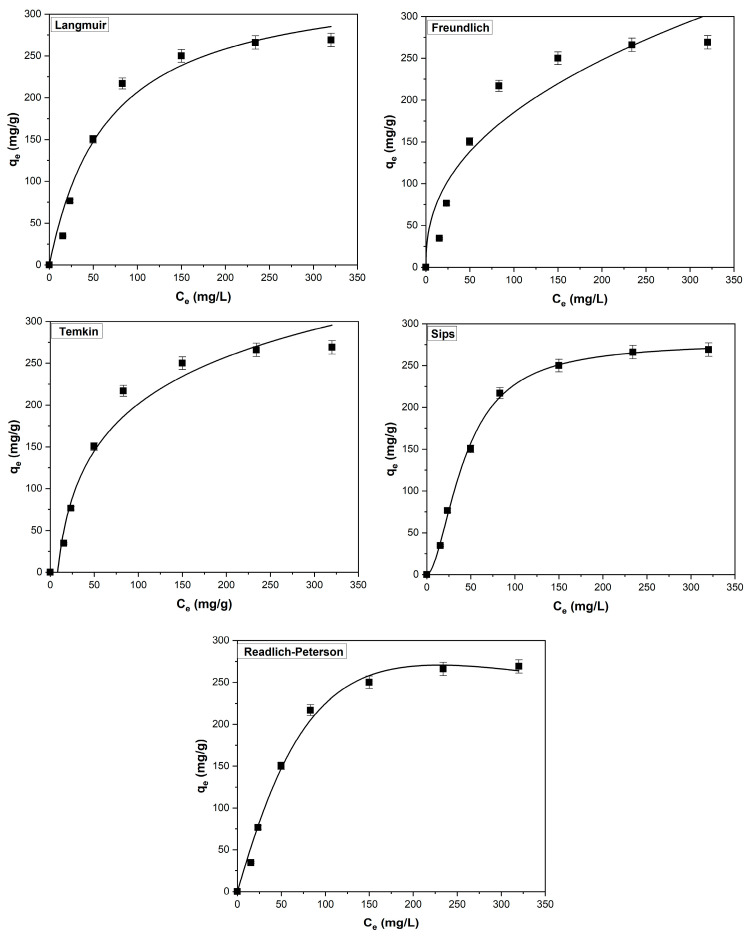
Experimental data points and fitted equilibrium isotherm for the adsorption of methylene blue on the adsorbent derived from coltsfoot leaves (experimental conditions: pH = 6, contact time = 20 min, adsorbent dosage = 1 g/L, temperature = 295 K, ionic strength = 0 mol/L).

The adsorption equilibrium was analyzed by fitting the experimental data with the five isotherm models: Langmuir, Freundlich, Sips, Temkin, and Redlich–Peterson (Figure 6). Table 4 contains the constants of the isotherms, along with their respective error parameters. The data in the table suggest that the Sips isotherm provides the best fit for the process, as it exhibits the highest R^2^ value and the lowest values for SSE, χ^2^, and ARE.

This isotherm model merges aspects of both the Langmuir and Freundlich isotherms, making it suitable for describing adsorption on heterogeneous surfaces. It also addresses the limitations of the Freundlich model, particularly its inability to predict saturation at higher adsorbate concentrations. At elevated adsorbate levels, the Sips model approaches the monolayer adsorption typical of the Langmuir isotherm, whereas at lower concentrations, it aligns more closely with the Freundlich isotherm [32].

The scientific literature reports that this isotherm has characterized the adsorption process of methylene blue on similar adsorbent materials: sour-cherry leaves [20], bilberry leaves [21], *Leonurus cardiaca* L. biomass [51], and raspberry leaves [34].

Table 5 presents a comparison of adsorption capacities for the adsorption of methylene blue on different plant-leaf-derived adsorbents. The value obtained for the absorption capacity of the adsorbent obtained from coltsfoot leaves is higher than that of other similar materials presented in the scientific literature.

### 3.4. Influence of pH, Ionic Strength, and Adsorbent Dose on Adsorption Capacity

**Figure 7:**
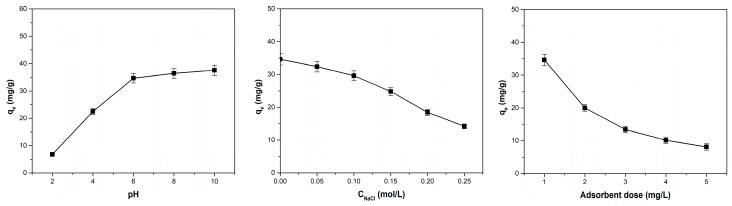
The effect of pH, ionic strength, and adsorbent dose on the adsorption capacity of the adsorbent material obtained from coltsfoot leaves.

The pH, ionic strength, and amount of adsorbent are key factors that have a substantial impact on the adsorption of cationic dyes. Figure 7 shows how these variables affect the uptake of methylene blue by the adsorbent obtained from coltsfoot leaves.

It can be seen that increasing the pH has a beneficial effect on the adsorption capacity, while increasing the ionic strength and adsorbent dosage has an unfavorable effect. Improved adsorption occurs at higher pH than pH_PZC_ (6.02) due to the stronger electrostatic attraction between the cationic methylene blue dye and the negatively charged surface of the adsorbent. At lower pH values, the surface becomes positively charged, leading to the opposite effect between adsorbent surface and cationic dye. The adsorption capacity varies slightly over the pH range 6 to 10, showing minimal increase. This suggests that electrostatic interactions are not the sole mechanism involved in the dye adsorption process [29].

Increasing ionic strength reduces adsorption capacity because sodium ions compete with dye cations for the occupancy of available adsorption sites on the adsorbent surface. By increasing the adsorbent dose, the reduction in adsorption capacity is probably caused by the fact that although the number of sites available for adsorption increases, many of these sites remain unsaturated, to which is added the probability of agglomeration of adsorbent material particles [20,21,34].

The same effect of pH, ionic strength, and adsorbent dose on adsorption capacity was recorded for the adsorption of methyl blue dye on sour-cherry leaves [20], bilberry leaves [21] phoenix tree leaves [28], lotus leaf [29], and raspberry leaves [34].

### 3.5. Thermodynamic Study

**Figure 8:**
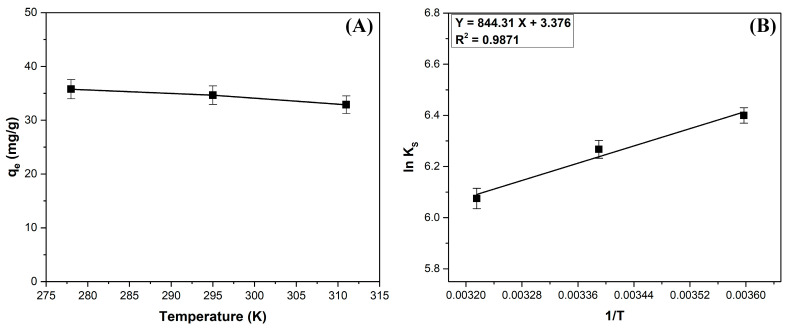
Thermodynamic data: (**A**) Influence of temperature on adsorption capacity, (**B**) Plot of ln KS vs. 1/T for methylene blue adsorption.

The influence of temperature on the adsorption capacity is illustrated in Figure 8A. It can be seen that temperature only slightly influences the process, the variation in adsorption capacity with temperature being practically extremely reduced.

Based on experimental data recorded at 278, 295, and 311 K, the values of the thermodynamic parameters were determined (Figure 8B, Table 6). The negative values of the standard Gibbs free energy change (ΔG°) and standard enthalpy change (ΔH°) indicate that the process is spontaneous, favorable, and exothermic. A positive value of the standard entropy change (ΔS°) implies an increase in disorder at the solid–liquid interface [29,48]. Comparable findings were reported in other scientific articles investigating the adsorption of methylene blue on various materials, including *Salix babylonica* leaves [27], *Daucus carota* leaves [19], sour-cherry leaves [20], and *Typha angustifolia* L. leaves [23].

The values of the thermodynamic parameters show that the main mechanism involved in the retention of methylene blue on the adsorbed material obtained from coltsfoot leaves is physisorption (ΔG° range from 20 to 0 kJ/mol, ΔH° < 40 kJ/mol) [20,27], and van der Waals forces play a significant role in the process (ΔH° < 20 kJ/mol) [20,34].

### 3.6. Adsorption Process Optimization

The optimal values of the parameters affecting the efficiency of dye removal from water through adsorption were determined using the Taguchi method. This approach is particularly well-suited for such applications as it reduces the impact of the noise factors on the process. The Taguchi method is widely recognized for its ability to optimize processes without incurring additional costs. It enables the evaluation of the influence of multiple variables using a limited number of experimental runs, thereby enhancing overall process effectiveness. A key benefit of this method compared to others is its capacity to simultaneously optimize several parameters while extracting detailed insights from a minimal number of experiments [54,55].

The optimal conditions for methylene blue removal through adsorption were determined using a Taguchi (L27) orthogonal array. Six factors, at three levels, were examined to assess their impact on dye removal efficiency (Table 7).

The Taguchi method was employed to transform the experimental data into a signal-to-noise (S/N) ratio, which was then analyzed to assess the quality of the experiment and validate the results. In this context, “signal” represented the desired value (mean), while “noise” referred to the undesirable value (standard deviation) for the output characteristic [54,55]. The highest adsorption efficiency was chosen as the basis for evaluating the experimental outcomes, and thus, the “larger-the-better” criterion was applied to analyze the signal-to-noise ratio in the Taguchi approach. The experimental results obtained for methylene blue removal efficiency and the corresponding S/N ratios for each run are illustrated in Table 8.

Table 9 summarizes the results of the Taguchi method, based on the S/N ratio, indicating the ranking of each factor’s significance. Among the controllable factors, pH had the greatest influence on the process, whereas the temperature had the least effect. By correlating the data from Table 7 with those in Table 9, the optimal conditions for the adsorption process can be determined as follows: pH = 10, contact time = 40 min, adsorbent dose = 5 g/L, initial dye concentration = 200 mg/L, temperature = 278 K, and ionic strength = 0 mol/L.

The ANOVA results confirm the results obtained by the Taguchi method and indicate the same order of influence for the controllable factors and, in addition, establish the percentage contribution of each factor (Table 10).

**Figure 9:**
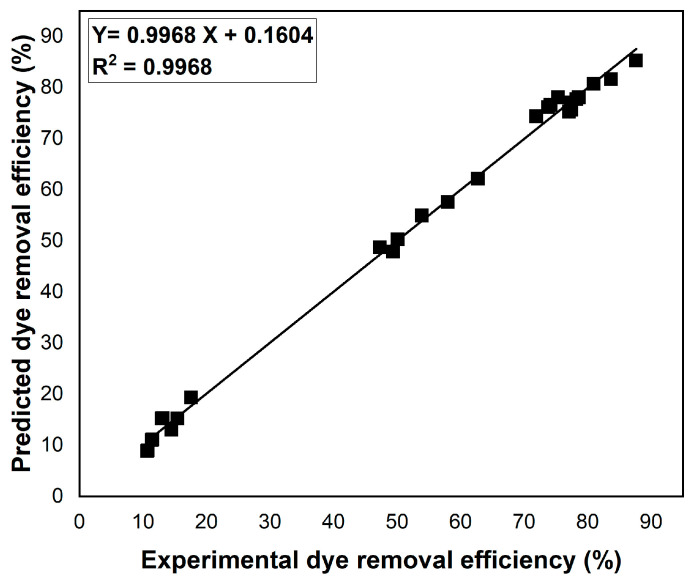
Correlation between experimental and predicted dye removal efficiency values.

The reliability of the predictions obtained by the Taguchi optimization approach was verified. A high degree of agreement was found between the experimental results and the predicted values for the dye removal efficiency (Figure 9). This was supported by a coefficient of determination (R^2^) value close to 1, demonstrating the predictive capacity of the model used for optimization.

## 4. Conclusions

In this study, the adsorption of methylene blue from aqueous solutions was studied using a new lignocellulosic adsorbent material obtained from mature coltsfoot (Tussilago farfara) leaves, after minimal processing of the leaves, without chemical or thermal treatment. The FTIR spectrum mainly highlights bands specific to functional groups in the structure of cellulose, hemicellulose, and lignin, and the color analysis confirms the color change in the adsorbent by retaining methylene blue on its surface. The Sips isotherm and the general kinetic model characterize the adsorption process, and the thermodynamic parameters indicate that the main mechanism involved in dye retention is physical adsorption. Optimal adsorption conditions were determined by the Taguchi method, which also indicated that among the controllable factors, pH had the greatest influence on the process, while temperature had the least effect. The ANOVA results confirmed the results obtained by the Taguchi method and indicated that the percentage contribution of the pH influence was 87.78% and that of the temperature was 0.16%. All results show that coltsfoot (*Tussilago farfara*) leaves are a very cheap, environmentally friendly, and effective adsorbent for the removal of methylene blue dye from aqueous solutions, its adsorption capacity of 278.1 mg/g being higher than other similar adsorbents.

## Figures and Tables

**Table 1:** FTIR spectrum bands and their assignments.

FTIR Bands (1/cm)	Assignment	Reference
3610	–OH stretch, free hydroxyl	[35]
3300	–OH stretching vibrations of cellulose, lignin, or hemicellulose	[36]
2924	–CH_2_ groups of cellulose, respectively	[37]
1680	C = C stretching	[38]
1550	amide II groups	[39,40]
1422	–C–H deformation in lignin	[41,42]
1282	–CH deformation in cellulose I and cellulose II	[43]
1057	C–O–C stretching of cellulose	[27,44]
698	aromatic out of plane C–H bending vibrations	[45,46]
542	C–H bend	[47]

**Table 2:** Equilibrium times for different adsorbents obtained from leaves, used for the adsorption of methylene blue.

Adsorbent	Equilibrium Time (min)	Reference
Humulus japonicas leaves	20	[18]
**coltsfoot leaves**	**20**	**This study**
*Daucus carota* leaves	30	[19]
sour-cherry leaves	40	[20]
bilberry leaves	40	[21]
*Magnolia grandiflora* leaves	60	[22]
*Typha angustifolia* leaves	60	[23]
*Platanus orientalis* leaf powder	70	[24]
*Ficcus Palmata* leaves	80	[25]
*Ginkgo biloba* leaves	100	[26]
*Salix babylonica* leaves	120	[27]
phoenix tree leaves	150	[28]
lotus leaf	150	[29]

**Table 3:** The calculated constants for tested kinetic models and associated error parameters.

Kinetic Model	Parameters	Values
Pseudo-first order	k_1_ (1/min)	0.48 ± 0.09
	q_e,calc_ (mg/g)	34.82 ± 2.71
	R^2^	0.9972
	χ^2^	0.09
	SSE	2.92
	ARE (%)	14.01
Pseudo-second order	k_2_ (1/min)	0.021 ± 0.006
	q_e,calc_ (g/mg·min)	37.21 ± 2.98
	R^2^	0.9887
	χ^2^	0.43
	SSE	12.13
	ARE (%)	3.39
Elovich	a (g/mg)	0.23 ± 0.05
	b (mg/g·min)	827 ± 141
	R^2^	0.9645
	χ^2^	1.42
	SSE	38.08
	ARE (%)	18.20
General order	k_n_ (1/min) (g/mg)^1/*n*^	8.98 ± 0.93
	q_n_ (mg/g)	19.91 ± 1.57
	*n*	3.34 ± 0.67
	R^2^	0.9979
	χ^2^	0.04
	SSE	0.69
	ARE (%)	0.94
Avrami	k_AV_ (1/min)	0.84 ± 0.12
	q_AV_ (mg/g)	34.82 ± 3.24
	n_AV_	0.57 ± 0.11
	R^2^	0.9972
	χ^2^	0.09
	SSE	2.92
	ARE (%)	14.01

**Table 4:** The adsorption isotherms models constants and the corresponding error functions.

Isotherm Model	Parameters	Value
Langmuir	K_L_ (L/mg)	0.015 ± 0.002
	q_max_ (mg/g)	344.4 ± 24.58
	R^2^	0.9764
	χ^2^	20.80
	SSE	2143
	ARE (%)	12.57
Freundlich	Kf (mg/g)(L/mg)^1/*n*^	26.64 ± 3.47
	1/*n*	0.42 ± 0.07
	R^2^	0.9134
	χ^2^	56.08
	SSE	7302
	ARE (%)	20.54
Temkin	K_T_ (L/mg)	0.11 ± 0.03
	b (kJ/g)	30.14 ± 4.9
	R^2^	0.9730
	χ^2^	13.85
	SSE	2228
	ARE (%)	10.89
Sips	Q_sat_ (mg/g)	278.1 ± 17.64
	K_S_ (L/mg)	0.0012 ± 0.0002
	*n*	1.78
	R^2^	0.9986
	χ^2^	1.22
	SSE	115
	ARE (%)	3.63
Redlich–Peterson	K_RP_ (L/g)	3.56 ± 0.78
	a_RP_ (L/mg)	0.0006 ± 0.0001
	β_RP_	1.49 ± 0.14
	R^2^	0.9934
	χ^2^	7.54
	SSE	602
	ARE (%)	7.41

**Table 5:** Adsorption capacities for the adsorption of methylene blue on different plant-leaf-derived adsorbents.

Adsorbent	Adsorption Capacity (mg/g)	Reference
*Ficcus Palmata* leaves	6.89	[25]
Ginkgo biloba leaves	48.07	[26]
*Salix babylonica* leaves	60.90	[27]
*Daucus carota* leaves	66.50	[19]
phoenix tree leaves	80.90	[28]
*Typha angustifolia* leaves	106.7	[23]
*Platanus orientalis* leaf	114.9	[24]
*Humulus japonicas* leaves	145.6	[18]
*Magnolia grandiflora* leaves	149.2	[22]
sour-cherry leaves	168.6	[20]
bilberry leaves	200.4	[21]
lotus leaf	221.7	[29]
raspberry leaves	244.6	[34]
**coltsfoot leaves**	**278.1**	**This study**
guava leaf	295.0	[53]

**Table 6:** Thermodynamic parameters for the adsorption of methylene blue on different plant-leaf-derived adsorbents.

ΔG (kJ mol^−1^)			ΔH (kJ mol^−1^)	ΔS (J mol^−1^ K^−1^)
278 K	297 K	311 K		
−14.79	−15.27	−15.70	−0.84	3.37

**Table 7:** The six three-level controllable factors that were used to evaluate the effect on dye removal efficiency.

Factor	Level 1	Level 2	Level 3
pH	2	6	10
Contact time (min)	5	20	40
Initial dye concentration (mg/L)	50	200	500
Adsorbent dose (g/L)	1	3	5
Temperature (K)	278	295	311
Ionic strength (mol/L)	0	0.15	0.25

**Table 8:** The L27 orthogonal array experimental setup, along with the dye removal efficiency values obtained from the experiments and the associated S/N ratios for each trial.

pH	Time (min)	Initial Dye Concentration (mg/L)	Adsorbent Dose (mg/L)	Temperature (K)	Ionic Strength (mol/L)	Dye Removal Efficiency (%)	S/N Ratio
2	5	50	1	278	0	13.11	22.35
2	5	50	1	295	0.15	11.50	21.21
2	5	50	1	311	0.25	10.79	20.66
2	20	200	3	278	0	17.61	24.91
2	20	200	3	295	0.15	15.45	23.77
2	20	200	3	311	0.25	14.49	23.22
2	40	500	5	278	0	12.99	22.27
2	40	500	5	295	0.15	11.40	21.13
2	40	500	5	311	0.25	10.69	20.57
6	5	200	5	278	0.15	77.07	37.73
6	5	200	5	295	0.25	73.81	37.36
6	5	200	5	311	0	78.25	37.86
6	20	500	1	278	0.15	49.39	33.87
6	20	500	1	295	0.25	47.30	33.49
6	20	500	1	311	0	50.14	34.00
6	40	50	3	278	0.15	77.45	37.78
6	40	50	3	295	0.25	74.18	37.40
6	40	50	3	311	0	78.64	37.91
10	5	500	3	278	0.25	57.98	35.26
10	5	500	3	295	0	62.75	35.95
10	5	500	3	311	0.15	53.93	34.63
10	20	50	5	278	0.25	81.00	38.16
10	20	50	5	295	0	87.67	38.85
10	20	50	5	311	0.15	75.34	37.54
10	40	200	1	278	0.25	77.34	37.76
10	40	200	1	295	0	83.73	38.45
10	40	200	1	311	0.15	71.95	37.14

**Table 9:** Response table for signal to noise ratios.

Level	pH	Time	Initial Dye Concentration	Adsorbent Dose	Temperature	Ionic Strength
1	22.24	31.45	32.43	31.00	32.24	32.51
2	36.38	31.98	33.14	32.32	31.96	31.65
3	37.09	32.27	30.14	32.39	31.51	31.55
Delta	14.85	0.82	3.00	1.40	0.73	0.96
**Rank**	**1**	**5**	**2**	**3**	**6**	**4**

**Table 10:** Contribution percentage of each controllable factor on the process.

	pH	Time	Initial Dye Concentration	Adsorbent Dose	Temperature	Ionic Strength	Errors
Contribution percentage (%)	87.78	1.19	7.84	2.20	0.16	0.52	0.31

## Data Availability

All the experimental data obtained are presented in the form of tables and/or figures in the article and in the Supplementary Materials.

## Supplementary Materials

The following supporting information can be downloaded at: https://www.mdpi.com/article/10.3390/polym17111549/s1, Table S1: Nonlinear equations of adsorption isotherms and kinetic models used to model experimental data; Table S2: Calculation formulas for the specific parameters R^2^, χ^2^, SSE, and ARE; Table S3: Equations for calculating thermodynamic parameters.
