# Supplementary material for: Adsorption of Methylene Blue onto Environmentally Friendly Lignocellulosic Material Obtained from Mature Coltsfoot (Tussilago farfara) Leaves
Source: Polymers (Basel). 2025 Jun 2;17(11):1549. doi: 10.3390/polym17111549 (PMC12156970; doi:10.3390/polym17111549)
Supplement: Supplementary file 1 [file polymers-17-01549-s001.zip › polymers-3672906-supplementary.pdf]

polymers

MDPI

Supplementary material

Adsorption of Methylene Blue onto Environmentally Friendly Lignocellulosic Material Obtained from Mature Coltsfoot (*Tussilago Farfara*) Leaves

Giannin Mosoarca [1], Cosmin Vancea [1], Simona Popa [1], Maria Elena Radulescu-Grad [2,*], Mircea Dan [1], Cristian Tanasie [3] and Sorina Boran [1,*]

[1] Faculty of Chemical Engineering, Biotechnologies and Environmental Protection, Politehnica University Timisoara, V. Parvan Bd. No. 6, 300223 Timisoara, Romania; giannin.mosoarca@upt.ro (G.M.); cosmin.vancea@upt.ro (C.V.); simona.popa@upt.ro (S.P.); mircea.dan@upt.ro (M.D.)

[2] Coriolan Dragulescu Institute of Chemistry, Romanian Academy, Mihai Viteazu Bd. No. 24, 300223 Timisoara, Romania

[3] National Institute of Research and Development for Electrochemistry and Condensed Matter (INCEMC), Dr. A. Paunescu Podeanu St., no.144, 300569 Timisoara, Romania; tase@incemc.ro

* Correspondence: mradulescugrad@acad-icht.tm.edu.ro (M.E.R.-G); sorina.boran@upt.ro (S.B.)

Table S1. Nonlinear equations of adsorption isotherms and kinetic models used to model experimental data.

Equilibrium isotherm	Equation
Langmuir	$q_e = \frac{q_m \cdot K_L \cdot C_e}{1 + K_L \cdot C_e}$
Freundlich	$q_e = K_F \cdot C_e^{1/n_F}$
Temkin	$q_e = \frac{R \cdot T}{b} ln(K_T \cdot C_e)$
Redlich-Peterson	$q_e = \frac{K_{RP} \cdot C_e}{1 + a_{RP} \cdot C_e^{\beta_{RP}}}$
Sips	$q_e = \frac{Q_{sat} \cdot K_S \cdot C_e^n}{1 + K_S \cdot C_e^n}$
**Kinetic models**	**Equation**
Pseudo-first-order	$q_t = q_e(1 - exp^{-k_1 \cdot t})$
Pseudo-second-order	$q_t = \frac{k_2 \cdot t \cdot q_e^2}{1 + k_2 \cdot t \cdot q_e}$
Elovich	$q_t = \frac{1}{a} ln(1 + a \cdot b \cdot t)$
General order	$q_t = q_n - \frac{q_n}{[k_n \cdot (q_n)^{n-1} \cdot t \cdot (n-1) + 1]^{1/1-n}}$
Avrami	$q_t = q_{AV}[1 - exp(-k_{AV} \cdot t)^{n_{AV}}]$

q_m, Q_{sat} - maximum absorption capacities; K_L, K_F, K_T, K_S, K_{RP} - Langmuir, Freundlich, Temkin, Sips, Redlich-Peterson isotherms constants; $1/n_F$ - empirical constant indicating the intensity of adsorption; R - universal gas constant; T - absolute temperature; b - Temkin constant which related to the adsorption heat; a_{RP} - Redlich-Peterson isotherm constant; β_{RP} - Redlich-Peterson exponent (vary between 0 and 1); n - Sips isotherm exponent; q_t - dye amount adsorbed at time t; k_1, k_2, k_{AV}, k_n - rate constants of pseudo-first-order, pseudo-second-order, Avrami and general order models; q_e, q_n, q_{AV} - theoretical values for the adsorption capacity; a - desorption constant of Elovich model; b - initial velocity; n_{AV} - fractional exponent; n - general order exponent

Table S2. Calculation formulas for the specific parameters R², χ², SSE and ARE.

Error parameter	Equation
R^2	$R^2 = 1 - \frac{\sum_{i=1}^{n}\left(y_{i,exp} - y_{i,mod}\right)^2}{\sum_{i=1}^{n}\left(y_{i,mod} - \overline{y_{i,exp}}\right)^2}$
SSE	$SSE = \sum_{i=1}^{n}\left(y_{i,exp} - y_{i,mod}\right)^2$
χ^2	$\chi^2 = \sum_{i=1}^{n}\frac{\left(y_{i,exp} - y_{i,mod}\right)^2}{y_{i,mod}}$
ARE	$ARE = \frac{100}{n}\sum_{i=1}^{n}\left\lvert\frac{y_{i,exp} - y_{i,mod}}{y_{i,mod}}\right\rvert$

$y_{i,exp}$ - experimental value; $y_{i,mod}$ - modeled value; $\overline{y_{i,exp}}$ - mean values, n - total amount of information, n_p - model parameters number

Table S3. Equations for calculating thermodynamic parameters.

Thermodynamic parameters	Equation
Standard Gibbs free energy change	$\Delta G^0 = -R \cdot T \cdot lnK_L$
Standard enthalpy change<br>Standard entropy change	$lnK_L = \frac{\Delta S^0}{R} - \frac{\Delta H^0}{R \cdot T}$

R - universal gas constant; K_L - Langmuir constant; T - absolute temperature
